# Polymorphism and protein expression of MUTYH gene for risk of rheumatoid arthritis

**DOI:** 10.1186/s12891-017-1437-0

**Published:** 2017-02-07

**Authors:** Shih-Yin Chen, Hsin-Han Chen, Yu-Chuen Huang, Shih-Ping Liu, Ying-Ju Lin, Sui-Foon Lo, Yuan-Yen Chang, Hui-Wen Lin, Chung-Ming Huang, Fuu-Jen Tsai

**Affiliations:** 1 School of Chinese Medicine, China Medical University, Taichung, 404 Taiwan; 2 Genetics Center, Department of Medical Research, China Medical University Hospital, Taichung, 404 Taiwan; 3 Division of Plastic and Reconstructive Surgery, China Medical University Hospital, Taichung, 404 Taiwan; 4 Division of Immunology and Rheumatology, Department of Internal Medicine, China Medical University Hospital, Taichung, 404 Taiwan; 5 Center for Neuropsychiatry, China Medical University Hospital, Taichung, 404 Taiwan; 6 Department of Physical Medicine and Rehabilitation, China Medical University Hospital, Taichung, 404 Taiwan; 7 Department of Microbiology and Immunology, and Institute of Microbiology and Immunology, School of Medicine, Chung Shan Medical University, Taichung, Taiwan; 8 Department of Optometry, Asia University, Taichung, 413 Taiwan; 9 Department of Pediatrics, China Medical University Hospital, Taichung, 404 Taiwan; 10 Department of Medical Genetics, China Medical University Hospital, Taichung, 404 Taiwan

**Keywords:** RA, MUTYH, SNPs, CNVs, Oxidative DNA damage. DAS28

## Abstract

**Background:**

We have previously described the association between rheumatoid arthritis (RA) prevalence and the two mutY Homolog (E. coli) (MUTYH) SNPs (rs3219463 and rs3219476) among the Taiwanese population. This present study will aim to elucidate whether the SNPs can alter the expression of EGFR in the progression of RA.

**Methods:**

The cohort study included 368 Taiwan’s Han Chinese RA patients and 364 healthy controls. Blood samples collected from the participants were analyzed to determine their serum MUTYH levels and to identify rs3219463 SNP of MUTYH from their genomic DNA.

**Results:**

Our data resulted in a statistically significant difference in genotype frequency distributions at rs3219463 for RA patients and controls (*p* < 0.0002). Also, the patients with G carrier at rs3219463 were less likely to suffer from painful joints (*p* < 0.006) and DAS28 scores (*p* < 0.003). Furthermore, the increase in serum level of MUTYH was also observed in RA patients (*p* < 0.005).

**Conclusions:**

Our study showed that RA is associated with rs3219463 SNP in EGFR gene and an increased serum level of the MUTYH protein. These findings suggest MUTYH is worthy of further investigation as a therapeutic target for RA.

## Background

Rheumatoid arthritis (RA) is a chronic systemic inflammatory disease characterized by persistent leukocytes infiltration, suppressed synovial fluid leukocyte apoptosis and sustained synovial hyperplasia [1–5]. If not properly treated, chronic joint inflammation can lead to permanent joint damage, and thus, lead to deformity. Although the pathophysiological causes of RA are not fully known, oxidative stress-induced cellular deoxyribonucleic acid (DNA) damage has been implicated in its pathogenesis [6–9].

In human cells, oxidative DNA attacks are happening thousands and millions of times per cell per day [10]. We are concerned that these attacks may lead to unfavorable genetic alternations. Normal cellular metabolism and environmental factors such as radiation and UV light appear to be the endogenous and exogenous instigators of DNA damages [10]. For this, the evolutionary process has equipped us with sophisticated DNA repair systems to preserve genetic stability. The major mechanism that cells use to repair oxidative DNA lesions, such as 8-oxo-7,8-dihydroguanine (8-oxo-G) and numerous types of oxidative DNA base damage products, is known as the base excision repair (BER) system [11, 12]. BER is initiated by DNA glycosylases, which recognize and remove specific damaged or mispaired bases, forming AP sites. These AP sites are then cleaved by AP endonucleases to yield a 3' hydroxyl adjacent to a 5' deoxyribosephosphate (dRP). The resulting single-strand break can subsequently be repaired by either short-patch or long-patch BER [10, 13]. The association of base excision repair (BER) of oxidative DNA damage and oxidative stress with RA were described previously [6–9]. Here, we are interested in the enzymes involved in the BER pathway and its association with RA.

mutY Homolog (E. coli) (MUTYH) is a unique DNA glycosylase that proficiently recognizes and catalyzes the removal of a mispaired adenine (A) from A:8-oxo-G, a frequent DNA lesion estimated to emerge around 1000–7000 times per cell per day [14–16]. Thus MUTYH is a key factor for giving a way to the supreme BER that eventually restores the undamaged guanine (G) [17, 18]. Most DNA is packaged in chromosomes within the nucleus (nDNA), but it can also be found in the mitochondria (mtDNA). Both nDNA and mtDNA are essential for proper cellular functioning; they are needed to flawlessly replicate from one cell cycle to the next. This may be the reason why MUTYH, as a genome caretaker, is localized to both the nucleus and the mitochondria [19, 20]. Gene polymorphism and mutations that lead to MUTYH inactivation have been associated with many cancers and cancer-associated inflammatory responses [21–24]. A number of studies have demonstrated that oxidative DNA strand break, and oxidative stress are significantly increased in the mononuclear leukocytes, serum and synovial fluid from RA patients than healthy controls [6–9]. The presence of oxidized DNA in joints has also been linked with arthritis in both mice [25] and humans [26]. We have previously described the association of rheumatoid arthritis (RA) prevalence and two mutY Homolog (E. coli) (MUTYH) SNPs (rs3219463 and rs3219476) among the Taiwanese population [27]. In this present study, we increased the sample size of the cohort and aimed to find out whether the SNPs can alter the expression of MUTYH in the progression of RA.

## Methods

### Patient selection

The study subjects included 368 RA patients and 364 healthy subjects, which were recruited from China Medical University Hospital in Taiwan. Patients with RA, according to the revised America College of Rheumatology criteria [28, 29], were enrolled. Nephelometry detected the rheumatoid factor (RF), and values ≧ 30 IU/ml were defined as positive. The gender-age-matched healthy controls from the general population were selected by health examination. Blood samples were collected by venipuncture for genomic DNA isolation. Informed consent was obtained from all participants, and it was according to the guidelines approved by the local ethics committee.

### Genomic DNA extraction, genotyping and quantitation of MUTYH copy number

**Fig. 1:**
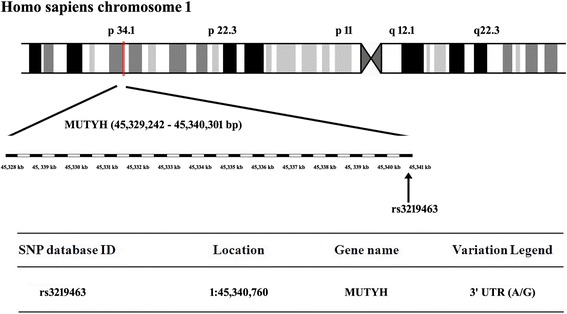
Map of MUTYH (rs3219463) located within Chromosome 1p34.1 region (45,329,242-45,340,301 bp)

Genomic DNA (gDNA) was prepared from peripheral blood using the genomic DNA kit from Roche. Polymerase chain reaction was used to identify the MUYTH rs3219463 polymorphism (Fig. 1). A pre-designed copy number assay (assay ID: Hs01177408_cn) from Applied Biosystems was used to quantify MUTYH copy number by TaqMan®Real-Time PCR. Primer sequences, reaction components, thermal cycling profiles and identification of various MUTYH genotypes by restriction enzymatic digestion and gel electrophoresis are all provided in the supplementary section.

### Quantitative determination of MUTYH in serum

Serum samples were diluted 1/200 in dilution buffer to be in the range of standard curve and then directly quantified by enzyme linked immunosorbent assay (Cat No. 30–7110, ALPCO Diagnostics, USA). The human MUTYH in the serums samples were bound to monoclonal mouse antibodies against human MUTYH, which were immobilized on the surface of the microtiter plates. After a washing step, the quantification of bound human MUTYH was carried out by adding a rabbit anti-human MUTYH antibody. Detection of the bound rabbit antibody was performed by a peroxidase labeled goat anti-rabbit antibody. The amount of converted substrate was directly proportional to the amount of bound human MUTYH and was photo metrically determined at 450 nm.

### Statistical analysis

The genotypic and allelic frequencies of MUTYH rs3219463 SNP for the RA patients and controls were compared using the chi-square test. When one cell had an expected count of <1 or >20% of the cells had an expected count of < 5, Fisher’s exact test was used. Results were considered statistically significant when *p* values less than 0.05. The odds ratios (OR) were calculated from the genotypic frequency and allelic frequency with a 95% confidence interval (95% CI) for the MUTYH rs3219463 SNP. The statistical analysis was performed by using SPSS version 11. The Mann–Whitney U test and Kruskal-Wallis method were used for non-parametric comparisons, and Student’s t test was used for parametric comparisons [30].

## Results

### Genotypic and allelic frequency distributions of MUTYH rs3219463 SNP among Taiwan’s Han Chinese population

The genotypic and allelic frequency distributions of rs3219463 SNP in the MUTYH gene were summarized in Table 1. The Hardy-Weinberg model was used to describe and predict genotype and allele frequencies in our study. We observed G allele to be the major allele in the population, regardless of whether they were in the patient group or the control group – at rs3219463 SNP site, the G allele frequencies were 57.6% (424 out of 736) for patients and 67.2% (489 out of 728) for the controls. By comparing the genotypic distributions between RA patients and healthy controls, our data indicated that individuals who carried GG or GA (G carrier) at rs3219463 SNP site are statistically significantly at a lower risk for developing RA (*p* < 0.0002).

**Table 1:** Genotypic and allelic frequency of MUTYH-437 (rs3219463) genetic polymorphism in the RA patients and controls

	RA patient	Control	OR (95% CI)	*p value*
MUTYH-437 (rs3219463)	*n* = 368 (%)	*n* = 364 (%)		
AA	54 (14.7)	47 (12.9)	1.80 (1.14-2.84)	6E-06 *
AG	204 (55.4)	145 (39.8)	2.20 (1.60-3.03)	
GG	110 (29.9)	172 (47.3)	Ref	
Allelic frequency				
Allele A	312 (42.4)	239 (32.8)	1.51 (1.22-1.86)	0.0002 *
Allele G	424 (57.6)	489 (67.2)	Ref	

*CI* confidence interval, *OR* odds ratio

* Statistically significant

### Normal MUTYH CNV in RA patients

Blood leukocytes gDNA samples were available from 227 RA patients and 223 healthy controls. For the remaining patients and controls, insufficient gDNA was collected to quantify MUTYH CNV. RA patients were not associated with abnormal MUTYH CNVs (Table 2). All patients and controls had 2 copies of the MUTYH gene present in their genome.

**Table 2:** Distribution of MUTYH CNV in the RA patients and controls

	RA patient	Control	OR (95% CI)	Chi-Square	Fisher’s Exact test
	*n* = 227 (%)	*n* = 223 (%)		*p value*	*p value*
MUTYH CNV					
0, 1 or 3	0	0	---	---	---
2	227 (100%)	223 (100%)			

*CI* confidence interval, *OR* odds ratio

### Increased serum level of MUTYH in RA patients

**Fig. 2:**
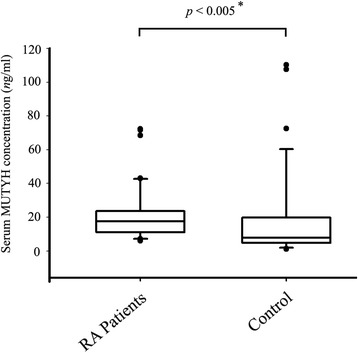
The expression level of MUTYH in the serum of RA patients and controls. p value was calculated by T-Test and Mann–Whitney U test. * means data is statistically significant

Serum levels of MUTYH were available from 40 RA patients and 38 healthy controls. For the remaining patients and controls, insufficient serum was collected to quantify MUTYH. There was a statistically significant increase in serum MUTYH concentration among RA patients (Fig. 2, *p* < 0.005). The mean MUTYH concentrations per milliliter of serum samples from RA patients and healthy controls are as follows: 21.62 ± 16.53 ng/mL for RA patients and 19.87 ± 27.47 for healthy controls. RA patients had 8.8% higher serum MUTYH levels than their age-, gender- and race-matched healthy controls.

### Classification of functional capacity and measurement of joint changes in RA patients carry risk- and non-risk-associated MUTYH SNP at rs3219463 site

RA patients can be categorized into four groups based on their functional capacity assessment. Class 1 patients can do all the usual activities without help. Class 2 patients can also do most of the normal activities despite feeling uncomfortable and thus limiting their mobility of one or more joints. Class 3 patients can do only a few tasks, and they are unable to work or they may not take care of themselves very well. Class 4 patients rely solely on others to take care them. There were no statistically significant difference between the RA-risk-associated group (AA at rs3219463) and the RA-non-risk-associated group (G carriers at rs3219463) in regards to the various RA diagnosis classes (Table 3). For the assessment of joint pain and joint stiffness, those RA patients who are G carriers at rs3219463 are less likely to have painful joints (Table 3, *p* < 0.01). We also performed quantitative joint examinations for our RA patients: the golden standard 66 swollen/68 tender joint counts (SJC66/TJC68), the disease activity score in 28 joints (DAS28), patient global assessment of disease activity (PtGA) and provider global assessment of disease activity (PrGA) were investigated among our RA patients. In our cohort, RA patients who are G carriers at rs3219463 showed statistically significant lower DAS 28 value (Table 3, *p* < 0.01) than the ones who carried AA at rs3219463. In addition, although the data was not statistical significance, those RA patients who were G carriers at rs3219463 tend to have lower SJC 66, TJC66, PtGA and PrGA values than those who carry AA at rs3219463 (Table 3).

**Table 3:** Classification of functional capacity and measurement of joint changes in RA patients

	RA patients		Chi-Square ^a^ *p* value	Fisher’s Exact test ^a^ *p* value
	Genotype at rs3219463			
	G carrier - n (%)	AA - n (%)		
RA Diagnosis class				
I	88 (75.2)	13 (56.5)	0.315	0.199
II	19 (16.2)	6 (26.1)		
III	8 (6.8)	3 (13.0)		
IV	2 (1.7)	1 (4.3)		
	RA patients		T-Test ^a^ *p* value	Mann–Whitney U test ^a^ *p* value
	Genotype at rs3219463			
	G carrier - n (mean ± SD)	AA - n (mean ± SD)		
MUTYH serum level ^b^	27 (20.98 ± 16.51)	13 (23.07 ± 17.16)	0.714	0.593
Joint pain	135 (31.93 ± 24.33)	28 (45.89 ± 25.46)	0.007 *	0.006 *
Joint stiffness	102 (0.62 ± 0.97)	19 (0.58 ± 0.72)	0.871	0.540
SJC 66 ^b^	142 (1.96 ± 2.69)	26 (2.81 ± 3.18)	0.156	0.250
TJC 68 ^b^	142 (3.83 ± 4.07)	26 (6.69 ± 7.06)	0.055	0.075
DAS 28	133 (3.88 ± 1.2)	25 (4.63 ± 1.23)	0.005 *	0.003 *
PtGA ^b^	135 (39.65 ± 26.72)	27 (50.48 ± 27.82)	0.058	0.052
PrGA ^b^	142 (23.73 ± 17.73)	27 (32.78 ± 23.38)	0.065	0.078

^a^ Nonparametric test

^b^ Mean value is lower in the G carrier / RA-non-risk-associated but data does not reach statistical significance

* Statistically significant

## Discussion

Rheumatoid arthritis (RA) can affect any joint in the body; the severity varies from person to person. Although the exact causes of RA are unknown, oxidative stress-induced DNA damage plays a big part. We performed a candidate gene study to investigate the association of MUTYH genetic variants with their expression and disease severity in RA patients. MUTYH was chosen as a candidate gene because MUTYH polymorphisms and mutations have been associated with various cancers and cancer-linked inflammatory responses [21–25]. RA is characterized by proliferative and invasive synovial fibroblasts in the synovium, a cell population with properties similar to cancer cells [1–5]. We initially tested the association of four SNPs (rs3219463, rs3219476, rs3219489 and rs3219493; data not shown) tagging MUTYH with the incidence of RA in a Taiwan Han Chinese cohort study. The MUTYH G to A polymorphism at rs3219463 SNP site was identified as a susceptible allele in relation to RA prevalence. Then, we carried out several follow-up studies that gave us a more detailed picture of MUTYH rs3219463 polymorphism and its association with RA severity.

The present study extends our earlier publication in 2015 on SNP rs3219463 of MUTYH in Taiwan-Chinese RA [27]. The sample sizes of RA increased from 192 to 368, and healthy control subjects from 192 to 364. As a follow-up study, the sample sizes for RA patients and controls were still relatively small. It would have been more impactful if the sample size for each group was greater than 500 samples. Currently, compared with other studies using Asian individual groups, we have the biggest sample size RA research. Here, we not only reporting the characterization of a SNP and potential copy number variation of MUTYH in rheumatoid arthritis patients, but also the MUTYH protein levels in RA patient’s serum. Our results indicate a significantly higher level of MUTYH in patients than in controls. To our knowledge, this is the first report on MUTYH serum protein levels in RA patients. And we think that is a very important information in RA development.

MUTYH is a DNA glycosylase that recognizes and excises mispaired adenine (A) bases from the DNA backbone. Post-translational phosphorylation of MUTYH is particularly important to grant itself the unique glycosylase activity on A:8-oxo-G and A:G mispairs [18, 31]. This enzyme, found in either nucleus or mitochondria, predominantly removes A from A:8-oxo-G mispairs under physiological salt concentrations [17]. As a genome caretaker that defends genome integrity, MUTYH is ubiquitously expressed and its activity is directed to newly synthesized DNA strands [32]. When MUTYH function is compromised, A:8-oxo-G mispairs on the newly synthesized DNA are not fixed, CG → AT transversion mutations can be generated in the next round of replication. Accumulations of deleterious mutations in normal cells ultimately transform them into cancer cells [33]. MUTYH-associated polyposis (MAP) and colorectal cancer (CRC) are the well-characterized hereditary conditions related to MUTYH mutations [23]. Indeed, the BER deficiency associated with MUTYH inactivation can happen to any cell type and affect any gene. The MAP tumor spectrum has been expanded to extra-colonic organs [22, 24, 34–37]. Synovial hyperplasia in RA is similar to a hyperplastic tumor, together with the fact that increased oxidative DNA damage and oxidative stress have been demonstrated in RA patients [6–9], we believed that MUTYH might have a role to play in RA pathology.

In this study, we examined the association of rheumatoid arthritis prevalence and the MUTYH rs3219463 polymorphism among Taiwan’s Han Chinese population. MUTYH rs3219463 SNP is located in 3’non-coding regions. This SNP has been linked with higher treatment-related mortality (TRM) risk and disease relapse in patients who have undergone allogeneic hematopoietic cell transplantation (HCT) [38]. Thyagarajan et al. proposed that lower BER activity can increase damage to normal tissues, resulting in higher cell death and thus increasing the risk of TRM [38]. MUTYH composes of localization sequence, a DNA binding domain, an adenine bind motif and several interaction domains for APE1, PCNA, RPA and MSH6 [39]. Various MUTYH isoforms differ in their 5’ sequence or first exon and they can be grouped into three categories, namely α, β and γ [40]. Studies have described MUTYH isoforms to differ in their 5’ sequence which will put them into different cellular compartments, either nucleus or mitochondria [19, 20, 40–42]. However, the distribution and functional statuses of different MUTYH isoforms to the different subcellular locations in different cell and tissue types are still not known. Indeed, examples of inter-isoformal regulation of their functional counterparts are held almost everywhere [43, 44]. Therefore, we propose to proceed further with the study of MUTYH isoform profiling and distribution in association with MUTYH SNPs.

Our pilot study has attempted to demonstrate whether MUTYH rs3219463 is associated with RA susceptibility and disease severity. There was a statistically significant increase in serum MUTYH concentration among RA patients. Unfortunately, we lacked samples to monitor the A/8oxoG base repair. Increased MUTYH expression may compensate for the loss of functional MUTYH isoform but further experiments are needed to validate this hypothesis. Also, a detailed questionnaire evaluated the joint damage (using joint pain, joint stiffness, SJC66/TJC68 and DAS28) and the global assessment of disease activity (using PtGA and PrGA) for our RA patients [45]. Although there were no statistically significant differences between various genotypes regarding RA function classes, indeed, RA patients who carried a potentially protective genotype (i.e. G carrier at rs3219463) had less severe disease symptoms – They were statistically significant in lower joint pain values and DAS scores. In addition, although the data did not reach statistical significance, RA patients who are G carriers at rs3219463 tend to have lower SJC 66, TJC66, PtGA and PrGA values than those who carried AA at rs3219463. Actually, the evaluation was somehow based on subjective analysis rather than on rigorous criteria. Pain level, stiffness level and tenderness level are subjective experiences of individuals. RA is a chronic illness and many patients with RA will suffer from depression. We cannot rule out that some patients may report physical inability and uncomfortableness were caused by psychological factors. In order to get statistically significant findings, a larger study group will be required to further verify the relationship between disease phenotypes and genotypes.

Both RF and anti-CCP are specific markers for RA because they are produced as part of the process that leads to joint inflammation in rheumatoid arthritis [46]. CRP is usually ordered along with ESR, and they measure how much inflammation is in the body [47]. Although ESR and CRP are not specific tests, flaring up of these values do indicate that you have inflammation somewhere in your body. The RA patients who are G carriers at rs3219463 tend to have lower mean RF and ESR values than the group who carried AA, but the difference did not reach statistical significance, possibly because of the small sample size of the subgroup analysis. The sample size is closely tied to statistical power. We have to admit that we have a small sample size in some subgroups, and sometimes we cannot get enough blood for both routine and RA-specific blood tests. Therefore, additional studies are needed to validate these results by using a larger cohort of RA patients.

Medications that can reduce joint inflammation, relieve pain and slow down joint damage are prescribed to treat or reduce the symptoms of RA. However, some of these medications can cause serious side effects [48, 49]. In addition, RA itself can increase the risk of developing certain cancers and organ dysfunctions in patients. These complications may affect serum MUTYH levels. We, thus, carefully documented the drug use patterns of each RA patient and no serious side effects were observed.

## Conclusions

In conclusion, our pilot study showed that RA is associated with rs3219463 SNP in MUTYH gene and increased serum level of the MUTYH protein. These findings suggest EGFR is a valuable therapeutic target in the treatment of RA, and thus, is worthy of further investigation.

## Abbreviations

95% CI: 95% Confidence interval
CNVs: Copy number variations
DAS: Disease activity score
MUTYH: mutY Homolog (E. coli)
RA: Rheumatoid arthritis
SJC66/TJC68: Swollen joint count (SJC66 joints) and tender joint count (TJC68 joints)
SNPs: Single nucleotide polymorphisms
